# Sinonasal Sarcoidosis Presenting as an Aggressive Nasal Destructive Lesion Mimicking Malignancy: A Diagnostic Challenge

**DOI:** 10.7759/cureus.97721

**Published:** 2025-11-25

**Authors:** Hafiz G Kamil, Badis A Harbawi, Laura Lu, Mohammed Haris, Elfatih Idris

**Affiliations:** 1 Respiratory Medicine, University Hospitals of North Midlands, Stoke-on-Trent, GBR; 2 Radiology, University Hospitals of North Midlands, Stoke-on-Trent, GBR; 3 Pathology, University Hospitals of North Midlands, Stoke-on-Trent, GBR

**Keywords:** atypical presentation of sarcoidosis, cancer mimic, differential diagnosis, pulmonary sarcoidosis, sino-nasal mass

## Abstract

Sarcoidosis is a multisystem granulomatous disease that commonly involves the lungs, with rare involvement of the sinonasal region. We present the case of a 64-year-old Asian female with a five-month history of painless nasal swelling and deformity. Imaging revealed an aggressive, fluorodeoxyglucose (FDG)-avid lesion involving the frontal sinuses, nasal septum, hard palate, and adjacent soft tissues, raising concern for malignancy such as lymphoma or squamous cell carcinoma. However, multiple biopsies from the nasal lesion, mediastinal lymph nodes, and a subcutaneous elbow nodule revealed non-necrotising granulomatous inflammation. A diagnosis of stage II pulmonary sarcoidosis with nasal and cutaneous involvement was confirmed through a multidisciplinary team review. This case highlights the diagnostic challenge posed by sinonasal sarcoidosis, which can mimic malignancy both clinically and radiologically, especially in the absence of systemic symptoms related to sarcoidosis. Early consideration of sarcoidosis in the differential diagnosis of destructive sinonasal lesions can help avoid unnecessary investigations and guide appropriate management.

## Introduction

Sarcoidosis is a multisystem granulomatous disease commonly involving the lungs and the mediastinum. Sarcoidosis may not only affect the lungs and mediastinum, but also any other organ, and extrapulmonary involvement is observed in about 30-40% of patients with sarcoidosis [1]. Even though predilection of the respiratory tract by sarcoidosis and extrapulmonary involvement of other organs is frequently observed, there is only limited data available regarding the frequency of sinonasal involvement [1]. Here, we present a diagnostically challenging case of sinonasal sarcoidosis presenting with an aggressive nasal deformity, initially suspected to be malignancy, to highlight the importance of considering sarcoidosis in the differential diagnosis of destructive sinonasal lesions, in conjunction with further relevant investigations.

## Case presentation

A 64-year-old Asian female with a background of type 2 diabetes mellitus was referred to the Ear, Nose, and Throat (ENT) team on a two-week wait pathway due to progressive nasal deformity. She reported a five-month history of painless nasal swelling without discharge, anosmia, facial numbness, or visual changes. Nasal endoscopy revealed no mucosal abnormalities.

**Figure 1:**
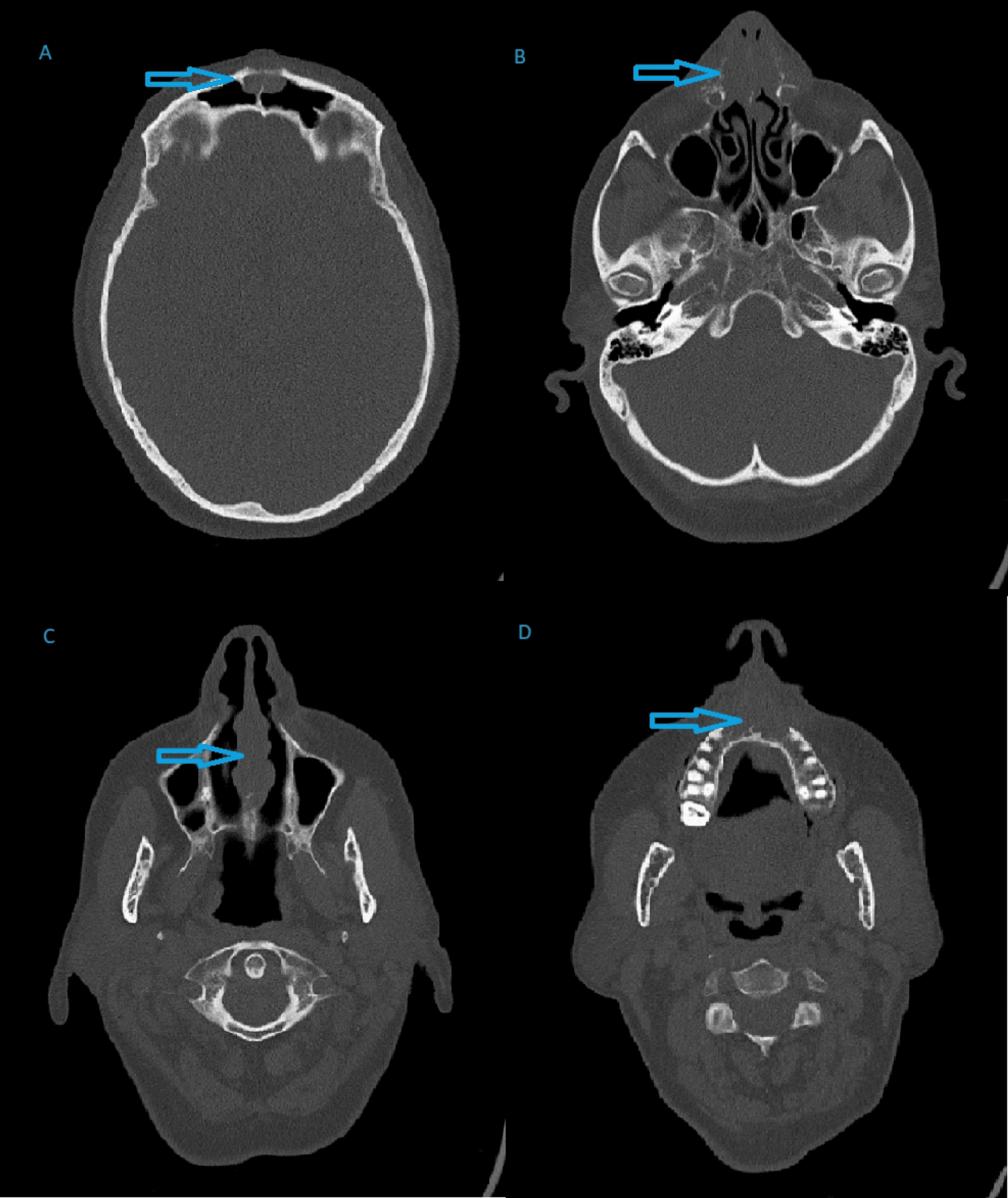
CT Scan in Sinonasal Sarcoidosis Case CT scan showing (A) a central frontal soft tissue lesion eroding the anterior wall of the frontal sinuses and extending to the adjacent subcutaneous tissue. (B) Further soft tissue thickening and bony erosions in the bridge of the nose and nasal bone with involvement of the distal nasolacrimal ducts and extension to the nasolabial folds. (C) Lobulated soft tissue mass involving the nasal septum inferiorly. (D) Erosion of the alveolar process of the maxillary bone with extension to the hard palate.

Computed tomography (CT) scan of the paranasal sinuses revealed a central frontal soft tissue lesion eroding the anterior wall of the frontal sinuses and extending into adjacent subcutaneous tissue. Additional soft-tissue lesions and bony erosions were noted in the nasal bridge, nasal bone, alveolar process of the maxilla, and hard palate, with a lobulated soft-tissue mass involving the inferior nasal septum and eroding through the hard palate. No septal perforation was seen. Paracentral nasal soft tissues were extending towards the nasolabial fold region bilaterally and involved the anteromedial aspect of the bilateral maxillary sinuses as well. Mild erosions of nasolacrimal ducts were seen (Figure 1).

**Figure 2:**
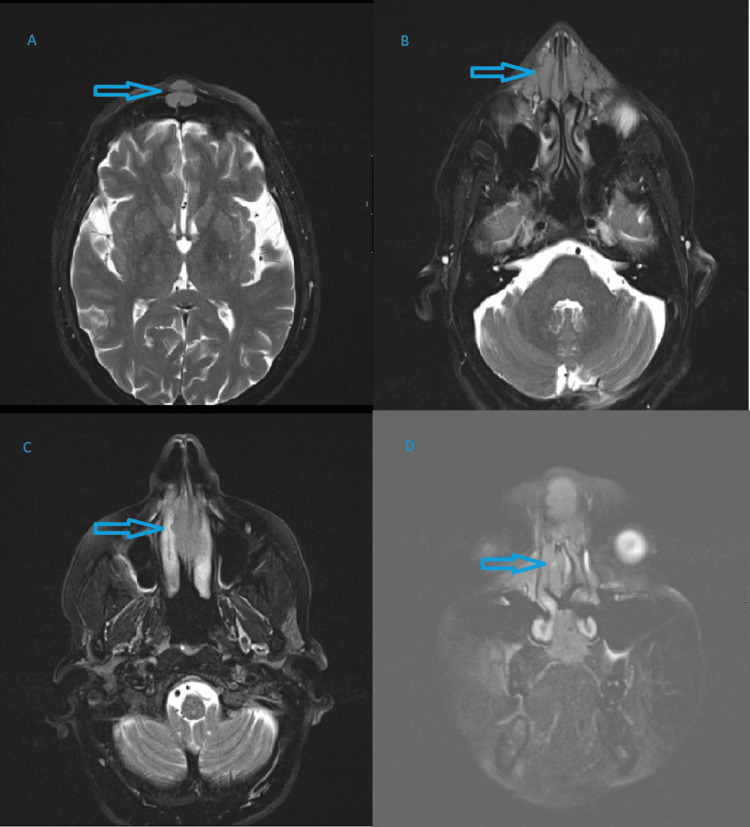
MRI Scan in Sinonasal Sarcoidosis Case MRI scan showing (A) axial T2 image showed a soft tissue lesion centred in the midline of the anterior frontal sinus wall. (B) Axial T2 image demonstrating soft tissue thickening involving the nasal bridge, nasal alae, anteromedial maxillary sinus wall and distal nasolacrimal ducts. (C) Axial T2 image showing a lobulated soft tissue lesion involving the nasal septum. (D) Coronal T2 fat-saturated image illustrating erosions of the alveolar process of the maxillary bone and a hard palate lesion.

**Figure 3:**
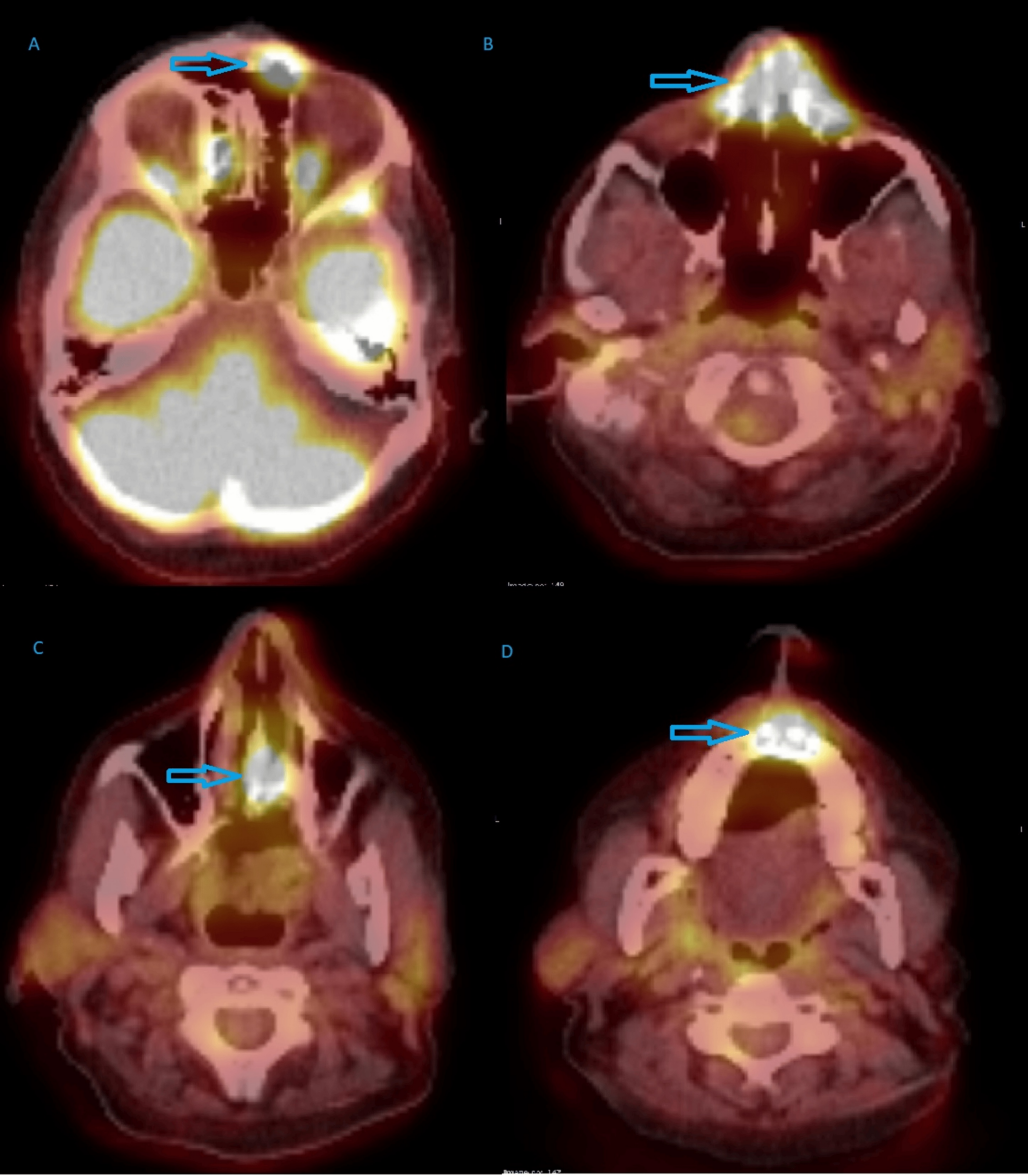
PET Scan in Sinonasal Sarcoidosis Case PET scan showing intense FDG-avid soft tissue involving (A) the central frontal sinuses anterior wall, (B) the nasal bone and cartilage/alae with extension to nasolabial folds, (C) the nasal septum, and (D) the alveolar process of the maxillary bone and hard palate.

Magnetic resonance imaging (MRI) of the paranasal sinuses added a comment of suspected tumour involving the above structures. Bilateral level IB rounded lymph nodes measuring about 12 mm in size were of concern. It was also reported as an aggressive pathology (Figure 2). Differentials were squamous cell carcinoma, metastatic disease or lymphoma, prompting a full-body positron emission tomography (PET) scan. It showed Intense fluorodeoxyglucose (FDG)-avid soft tissue involving the frontal sinuses, nose, nasal septum and extending up to the nasolabial fold, which was again typical of malignancy (SUVmax 13.6). Marked FDG avid multi-station mediastinal and bilateral hilar lymph nodes were suspicious, along with mildly FDG avid bilateral axillary, bilateral inguinal and bilateral external iliac lymph nodes (SUVmax 4.8). Mild FDG avid soft tissue nodules in the right forearm (SUVmax 3.5) and adjacent to the right elbow (SUVmax 8.2) were also suspicious, and the overall findings favoured lymphoma with metabolic stage 4 disease or less likely nasal squamous cell carcinoma (Figure 3).

Biopsy of the nasal mass showed non-necrotising granulomatous inflammation. Stains for acid-fast bacilli (AFB) and fungal organisms were negative. Simultaneous blood tests were arranged to identify the cause, which were reported as below (Table 1).

**Table 1:** Relevant Laboratory Results in Sinonasal Sarcoidosis Case

Blood test	Result	Reference range
Lymphocytes	1.14 10× 10⁹/L	1.15-4.0
Angiotensin converting enzyme	67.0 U/L	12.0-71.0
Estimated glomerular filtration rate (eGFR)	>90 mL/min/1.7	-
Adjusted calcium	2.45 mmol/L	2.2-2.6
Anti-myeloperoxidase (MPO) antibodies	<0.7 IU/mL	<3.5
Anti-proteinase 3 (PR3) antibodies	<0.3 IU/mL	<2.0
Anti-nuclear antibodies	Homogeneous 1:320 and nucleolar 1:320 patterns	-
Rheumatoid factor	29.3 IU/mL	0-20

**Figure 4:**
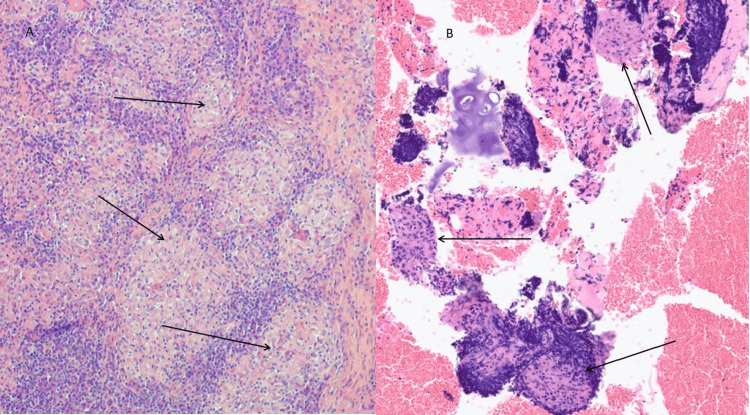
Histology in Sinonasal Sarcoidosis Case (A) Nasal biopsy and (B) endobronchial ultrasound-guided fine needle aspiration of mediastinal lymph node, both showing non-necrotising granulomatous inflammation.

The patient was referred to our respiratory services. She denied systemic symptoms such as cough, breathlessness, fatigue, rash, ocular, or joint involvement. A subcutaneous nodule was noted on the right elbow. Endobronchial ultrasound (EBUS)-guided biopsy of mediastinal lymph nodes and ultrasound-guided biopsy of the elbow nodule both confirmed non-necrotising granulomatous inflammation, consistent with sarcoidosis (Figure 4). AFB cultures were again negative.

She was subsequently referred to the Interstitial Lung Disease Multidisciplinary Team (ILD-MDT), confirming ‘early-stage II pulmonary sarcoidosis with nasal and cutaneous involvement’.

## Discussion

Sinonasal sarcoidosis is a rare manifestation of systemic sarcoidosis, occurring in less than 1% of cases, and often mimics malignancy clinically and radiologically. In our patient, the aggressive, FDG-avid sinonasal lesion with extensive bone destruction was initially suspected to be lymphoma or squamous cell carcinoma, highlighting the diagnostic challenge of this entity.

Kirsten et al. reported 12 cases of biopsy-proven sinonasal sarcoidosis, all in Caucasian patients and typically presenting with nonspecific nasal obstruction or crusting rather than destructive lesions [1]. Our case broadens the demographic spectrum, as it occurred in an Asian female and presented primarily with nasal deformity.

Aubart et al. reported that sinonasal involvement occurred in the course of previously known sarcoidosis in eight patients, whereas it preceded disease diagnosis in 12 patients. Among these 12 patients, four initially presented with strictly isolated sinonasal sarcoidosis, and eight had other associated signs related to sarcoidosis [2]. Our case aligns with the latter pattern, as the patient presented with nasal deformity as the initial and sole complaint, without systemic symptoms at the time of presentation. This emphasises that sinonasal sarcoidosis can not only precede systemic disease but also present as an isolated, aggressive lesion, making early recognition crucial to avoid misdiagnosis and inappropriate oncologic workup.

While Braun et al. noted elevated angiotensin converting enzyme (ACE) levels in most of their cases [3], our patient’s normal ACE value demonstrates that normal biochemical markers do not exclude the diagnosis. The intense FDG uptake seen here, though typical of malignancy, has also been described in sarcoidosis and may lead to false-positive PET interpretations.

Corticosteroids remain first-line therapy, and surgery is reserved for persistent obstruction or deformity. Mills et al. described a case requiring turbinate reduction [4].

This case underscores that sinonasal sarcoidosis may present as an isolated, aggressive, destructive lesion even in the absence of systemic features or elevated serum markers. Early tissue diagnosis and multidisciplinary evaluation are essential to avoid misdiagnosis and unnecessary oncologic interventions.

## Conclusions

This case highlights the diagnostic complexity of rare sinonasal sarcoidosis, especially when it presents with aggressive sinonasal destruction and lacks systemic symptoms. It underscores the importance of considering sarcoidosis as a differential in destructive nasal lesions, even though imaging was strongly suggestive of malignancy. Increased clinical awareness and a low threshold for considering sarcoidosis can facilitate earlier recognition and appropriate management.
